# Towards a Data-Driven Estimation of Resilience in Networked Dynamical Systems: Designing a Versatile Testbed

**DOI:** 10.3389/fnetp.2022.838142

**Published:** 2022-03-17

**Authors:** Tobias Fischer, Thorsten Rings, M. Reza Rahimi Tabar, Klaus Lehnertz

**Affiliations:** ^1^ Department of Epileptology, University of Bonn Medical Centre, Bonn, Germany; ^2^ Helmholtz Institute for Radiation and Nuclear Physics, University of Bonn, Bonn, Germany; ^3^ Department of Physics, Sharif University of Technology, Tehran, Iran; ^4^ Institute of Physics, Carl von Ossietzky University of Oldenburg, Oldenburg, Germany; ^5^ Interdisciplinary Center for Complex Systems, University of Bonn, Bonn, Germany

**Keywords:** resilience, coupled oscillators, complex networks, time series analysis, testbed, FitzHugh-Nagumo

## Abstract

Estimating resilience of adaptive, networked dynamical systems remains a challenge. Resilience refers to a system’s capacity “to absorb exogenous and/or endogenous disturbances and to reorganize while undergoing change so as to still retain essentially the same functioning, structure, and feedbacks.” The majority of approaches to estimate resilience requires exact knowledge of the underlying equations of motion; the few data-driven approaches so far either lack appropriate strategies to verify their suitability or remain subject of considerable debate. We develop a testbed that allows one to modify resilience of a multistable networked dynamical system in a controlled manner. The testbed also enables generation of multivariate time series of system observables to evaluate the suitability of data-driven estimators of resilience. We report first findings for such an estimator.

## 1 Introduction

The term resilience is commonly used to describe the ability of a system to return to its normal condition after its state has been perturbed. It is closely related to the notion of *local* stability (Lyapunov, 1892; Holling and Goldberg, 1971). When dealing with adaptive dynamical systems, the *nonlocal stability* concept of *ecological resilience* is increasingly employed throughout a number of scientific disciplines. With this concept, resilience refers to the system’s capacity “to absorb exogenous and/or endogenous disturbances and to reorganize while undergoing change so as to still retain essentially the same functioning, structure, and feedbacks” (Walker et al., 2004; Barabási and Posfai, 2016; Schoenmakers and Feudel, 2021). Despite the wide use of this concept, there is by now no commonly accepted method to assess resilience. Rather, a plethora of different indicators of resilience were proposed which are often not directly comparable [for an overview, see Meyer (2016) and Quinlan et al. (2016)]. Moreover, the vast majority of indicators of resilience require precise knowledge of the governing equations of motion and are thus of only limited value for the analysis of empirical data, such as for example time series of physiological observables of the human organism (Lehnertz et al., 2020; Romero-Ortuño et al., 2021).

Among the few data-driven indicators of resilience (or loss thereof) are the ones related to the concept of critical slowing down [see for example Scheffer et al. (2009); Dai et al. (2012); Lenton et al. (2012); Dakos et al. (2015); Scheffer et al. (2018)], namely lag-1 autocorrelation and variance (or other higher-order statistical indicators) estimated from time series of appropriate system observables. The suitability of these indicators has been extensively investigated with a variety of domain-specific models (see for example Weinans et al. (2021) and references therein). Nevertheless, a number of field applications indicate that these indicators are not always reliable (Ditlevsen and Johnsen, 2010; Boettiger and Hastings, 2013; Clements et al., 2019; Diks et al., 2019; Wilkat et al., 2019; Marconi et al., 2020; Hagemann et al., 2021; van der Bolt et al., 2021). In part, this can be attributed to the fact that the assumed mechanism behind critical slowing down (bifurcation-induced tipping) may be too simplistic for many natural systems which calls for data-driven indicators of resilience related to other transition scenarios (Kuehn, 2011; Ashwin et al., 2012; Kuehn et al., 2015; Ritchie and Sieber, 2017; Vanselow et al., 2019; O’Keeffe and Wieczorek, 2020; Hernández-Navarro et al., 2021; Schoenmakers and Feudel, 2021).

Another, more recently proposed, fully data-driven indicator of resilience – dynamical resistance (Rings et al., 2019) – takes into account that the dynamics of some elementary unit of a *N*-dimensional networked dynamical system can be described by its self-dynamics as well as by interactions with other units:

$${\dot{x}}_{i}=f\left(x_{i}\right)+\sigma \overset{N}{\underset{j=1}{\sum }}A_{ij}\;h\left(x_{j};x_{i}\right),$$ (1)

where ***f*** (***x***_*i*_) determines the self-dynamics of unit *i*. The coupling between units *i* and *j* is defined by a coupling strength *σ*, a coupling matrix ***A*** (binary adjacency matrix), and a coupling function ***h***, each of which can be time-dependent. With the ansatz of Rings et al. (2019), it is assumed that perturbations predominantly affect the *dynamical coupling structure* (second term on r.h.s of Eq. 1), so that a possible influence of a unit’s self-dynamics can be neglected. The authors proposed to approximate this structure with bivariate time-series-analysis techniques (Pikovsky et al., 2001; Kantz and Schreiber, 2003; Hlaváčková-Schindler et al., 2007; Marwan et al., 2007; Stankovski et al., 2017) in a time-resolved manner, and at the example of epileptic seizures in human brains, they demonstrated their approach to efficiently monitor dynamical resistance of a complex networked system prone to extreme events.

It is, however, not clear whether the assumptions underlying data-driven indicators of resilience are fully justified and whether indicators are generally applicable to estimate resilience of any real world system. An important step towards answering these questions is the development of a versatile testbed that would allow one to verify the reliability of data-driven estimators of resilience of networked dynamical systems. Here, we develop such a testbed that allows one to modify resilience of a multistable networked dynamical system in a controlled manner and to generate multivariate time series of system observables. We report first findings on the suitability of dynamical resistance as a data-driven indicator of resilience.

## 2 Setting up the Testbed

Before going into details, let us first list some basic requirements for a testbed to be versatile:

1 in order to simulate a multistable system, a testbed should allow for an adjustable number of system states but more than two;
2 in order to simulate normal (*desired*) and aberrant (*undesired*) states, a testbed should generate distinguishable dynamics for each state;
3 in order to allow data-driven indicators of resilience to reliably characterize different system states (including those with fragmented boundaries), waiting times within each state should be sufficiently long;
4 in order to allow for a number of modifications of the system’s resilience, a testbed should have sufficiently many control parameters.

### 2.1 Dynamics: From Oscillators to Networks of Oscillator Networks

For our testbed, we consider one of the most simple and widely studied excitable systems, namely the FitzHugh–Nagumo (FHN) oscillator (also known as Bonhoeffer–van der Pol oscillator) (van der Pol and van der Mark, 1928; Bonhoeffer, 1948; FitzHugh, 1961; Nagumo et al., 1962; Rocşoreanu et al., 2000), which is a prototypical model for excitable behavior in neural and cardiac nonlinear activities (Glass et al., 1991; Koch, 1999). The equations of motion for the FHN oscillator read:

$$\begin{array}{ll} \dot{x} & =x\left(a-x\right)\left(x-1\right)-y+I\\ \dot{y} & =bx-cy, \end{array}$$ (2)

with *x* and *y* denoting the (fast) excitatory and (slow) inhibitory dynamical state variables. Here *a*, *b*, and *c* are dimensionless control parameters: *a* and *c* are scaling parameters, and *b* controls the emergence of various dynamical regimes (such as tonic and phasic spiking or sub-threshold oscillations). *I* is the magnitude of stimulus current.

Networks of coupled, non-identical FHN oscillators can exhibit much richer dynamics depending on the coupling and the coupling topology. Apart from various synchronization phenomena (Chernihovskyi and Lehnertz, 2007; Omelchenko et al., 2015; Plotnikov et al., 2016; Masoliver et al., 2017; Ramlow et al., 2019; Gerster et al., 2020), such networks are capable of generating extreme-event-like phenomena (Ansmann et al., 2013; Karnatak et al., 2014; Rings et al., 2017; Saha and Feudel, 2017) and self-induced switching between multiple space-time pattern (Ansmann et al., 2016). The complexity of dynamics can further be enhanced, if one considers networks of networks of (diffusively coupled) FHN oscillators (Rydin Gorjão et al., 2018). Here the equations of motion for the *i*-th oscillator ( $i\in \left\{1,\ldots ,N_{\text{o}}\;\right\}$ ; *N*_o_ denotes the number of oscillators) in the *k*-th sub-network ( $\left(k,l\right)\in \left\{1,\ldots ,N_{\text{n}}\;\right\}$ ; *N*_n_ denotes the number of sub-networks) read:

$$\begin{array}{ll} {\dot{x}}_{i}^{\left(k\right)} & =x_{i}^{\left(k\right)}\left(a_{i}-x_{i}^{\left(k\right)}\right)\left(x_{i}^{\left(k\right)}-1\right)-y_{i}^{\left(k\right)}\\ \; & +\frac{C_{\text{w}}^{\left(k\right)}}{N_{\text{o}}\;-1}\overset{N_{\text{o}}\;}{\underset{j=1}{\sum }}S_{ij}\left(x_{j}^{\left(k\right)}-x_{i}^{\left(k\right)}\right)+\frac{1}{N}\overset{N_{\text{n}}\;}{\underset{l=1}{\sum }}C_{\text{b}}^{\left(k,l\right)}B_{kl}\overset{N_{\text{o}}\;}{\underset{j=1}{\sum }}\left(x_{j}^{\left(l\right)}-x_{i}^{\left(k\right)}\right)\\ {\dot{y}}_{i}^{\left(k\right)} & =b_{i}x_{i}^{\left(k\right)}-c_{i}y_{i}^{\left(k\right)}, \end{array}$$ (3)

where $C_{\text{w}}^{\left(k\right)}$ and $C_{\text{b}}^{\left(k,l\right)}$ denote the global coupling strengths within and between sub-networks *k* and *l*. For a given sub-network, $S\in {\left\{0,1\right\}}^{N_{\text{o}}\times N_{\text{o}}\;}$ denotes the symmetric adjacency matrix (*S*_*ij*_ = *S*_*ji*_ = 1, if oscillators *i* and *j* are coupled, else *S*_*ij*_ = *S*_*ji*_ = 0). The symmetric adjacency matrix $B\in {\left\{0,1\right\}}^{N_{\text{n}}\;\times \;N_{\text{n}}\;}$ characterizes the coupling structure between sub-networks. For the numerical integration of such large systems of differential equations, we use the python module jitcode (Ansmann, 2018).

**FIGURE 1:**
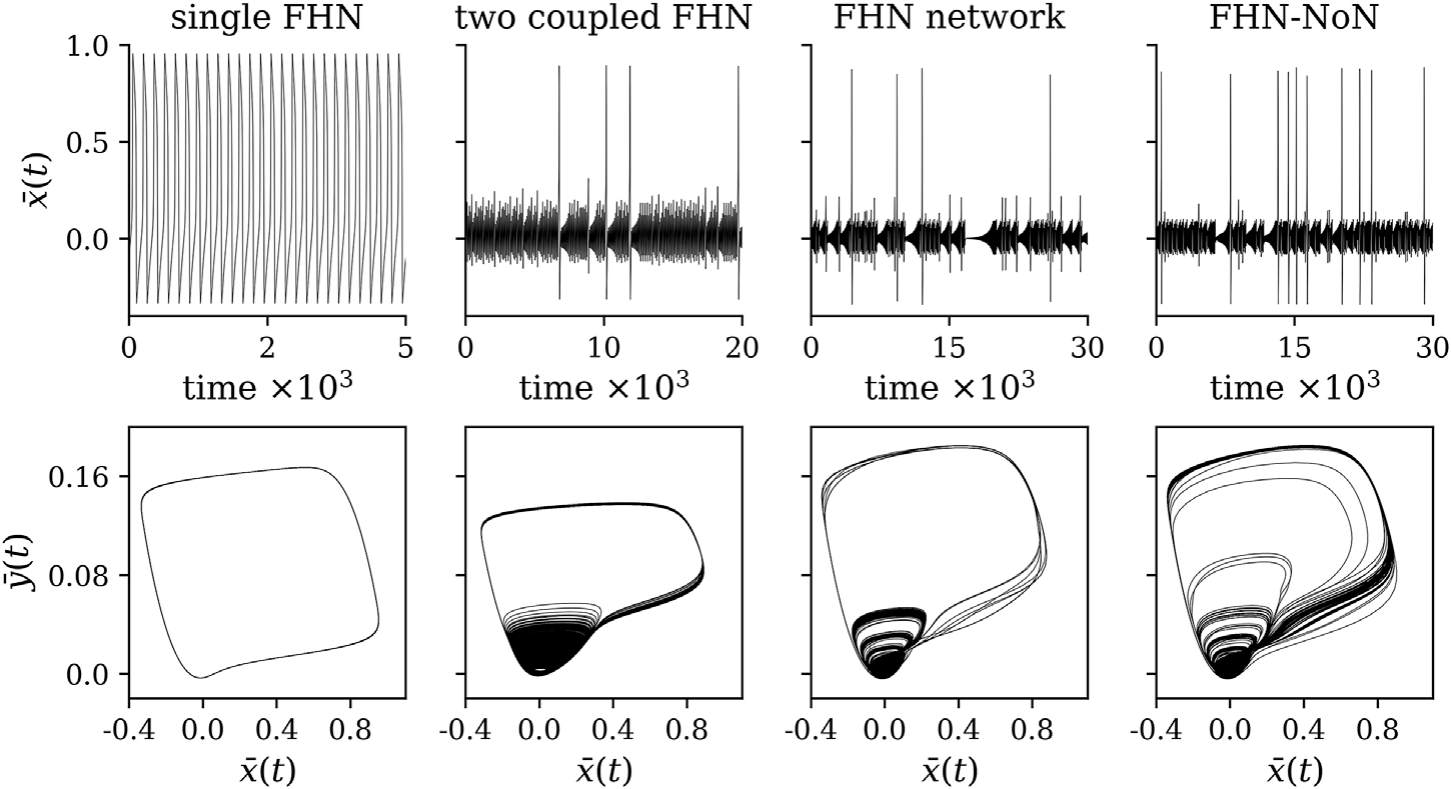
Exemplary time series and corresponding phase space representations of the dynamics of a single FHN oscillator (*β*_*c*_ = (0, 0, 0)), two coupled FHN (*β*_*c*_ = (0.130 8, 0, 0)), a fully connected network with 25 FHN oscillators (*β*_*c*_ = (0.115, 0, 0)), and a network of two networks with 50 FHN oscillators (*β*_*c*_ = (0.115, 0.215, 9.5 ⋅ 10^−5^)).

For our study, we consider *N*_n_ = 2 fully connected sub-networks, each consisting of *N*_o_ = 25 diffusively coupled non-identical FHN oscillators (FHN-NoN). The control parameters *a* and *c* are identical for all oscillators: *a*_*i*_ = *a* = − 0.027 6 *∀i* and *c*_*i*_ = *c* = − 0.02 *∀i*; parameter *b*_*i*_ is linearly distributed on $\left[0.006,0.014\right]$ to prevent immediate synchronization among oscillators for non-zero coupling strengths (cf. Ansmann et al. (2013) and Rydin Gorjão et al. (2018)). The set of coupling strengths $\beta_{c}=\left(C_{\text{w}}^{\left(1\right)},C_{\text{w}}^{\left(2\right)},C_{\text{b}}\right)$ is adjustable (note that we dropped the superscripts *k* and *k*| for the sake of readability). As an example, we show in Figure 1 time series of the averaged dynamical variables ${\bar{x}}^{\left(k\right)}\left(t\right)=\sum_{i=1}^{N_{\text{o}}\;}x_{i}^{\left(k\right)}\left(t\right)$ and ${\bar{y}}^{\left(k\right)}\left(t\right)=\sum_{i=1}^{N_{\text{o}}\;}y_{i}^{\left(k\right)}\left(t\right)$ of sub-network 1 for various *β*_*c*_, following Ansmann et al. (2013); Karnatak et al. (2014); Ansmann et al. (2016), and Rydin Gorjão et al. (2018). For the single FHN oscillator, we obtain the two Lyapunov exponents as *λ*_1_ = − 3.45 ± 6.14 ⋅ 10^−6^ and *λ*_2_ = − 2.89 ± 0.01 ⋅ 10^−1^. For two coupled FHN, the three largest Lyapunov exponents amount to *λ*_1_ = 4.07 ± 0.14 ⋅ 10^−3^, *λ*_2_ = 5.04 ± 12.06 ⋅ 10^−7^, and *λ*_3_ = − 7.00 ± 0.09 ⋅ 10^−2^. For the fully connected network with 25 FHN oscillators, we obtain for the three largest Lyapunov exponents *λ*_1_ = 5.91 ± 0.23 ⋅ 10^−3^, *λ*_2_ = 4.81 ± 13.01 ⋅ 10^−6^, *λ*_3_ = − 2.17 ± 0.05 ⋅ 10^−2^ and for the FHN-NoN *λ*_1_ = 6.42 ± 0.16 ⋅ 10^−3^, *λ*_2_ = 5.40 ± 8.85 ⋅ 10^−6^, *λ*_3_ = − 5.94 ± 0.78 ⋅ 10^−4^. These Lyapunov exponents (Benettin et al., 1980a,b) were derived from 20 realizations of the systems with different initial conditions.

### 2.2 Modeling a Multistable System

**FIGURE 2:**
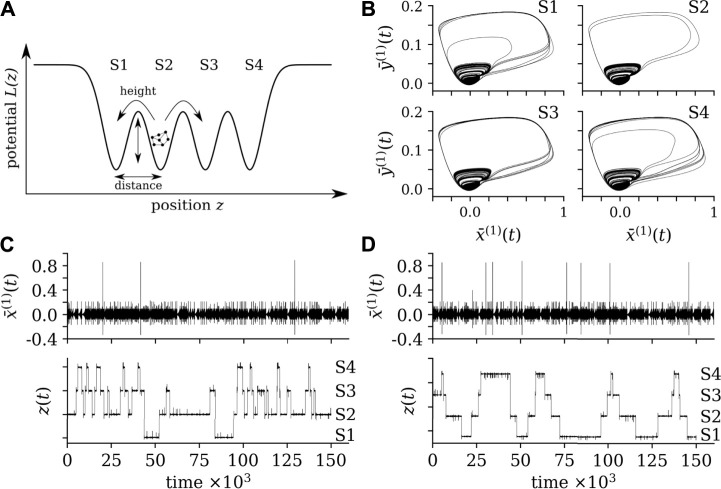
**(A)**: exemplary potential landscape with four system states. The potential landscape is driven by the mean field of FHN-NoN and, together, the system exhibits self-induced transitions. **(B)**: Exemplary phase space representations of the averaged dynamical variables for each state. **(C,D)**: Exemplary time series of the averaged dynamical variable ${\bar{x}}^{\left(1\right)}\left(t\right)$ of the FHN-NoN and of the dynamical variable *z*(*t*) of the potential landscape. In **(C)**, Γ(*t*) is a random sequence of + 1s and − 1s and in **(D)**, Γ(*t*) is chosen state-dependent such that the system evolves alternating from the first to the last state (S1 → S4: Γ(*t*) = + 1) and back (S4 → S1: Γ(*t*) = − 1). Settings of other parameters and conditions: random initial conditions for $x_{i}^{\left(k\right)}\times y_{i}^{\left(k\right)}\in \left[0,1\right]\times \left[0,1\right]$ and for *z* ∈ [ − 5, 5]; *a* ^G^ = [5.5, 5.5, 5.5, 5.5]; *μ* ^G^ = [ − 6, − 2, 2, 6]; ${\left(\sigma^{\text{G}}\right)}^{2}=\left[1.8,1.8,1.8,1.8\right]$ ; for all states: $C_{\text{w}}^{\left(1\right)}=0.115$ , for state S1: $C_{\text{w}}^{\left(2\right)}=0.116$ and *C*_b_ = 1.045 ⋅ 10^−5^; for state S2: $C_{\text{w}}^{\left(2\right)}=0.116$ and *C*_b_ = 1.425 ⋅ 10^−5^; for state S3: $C_{\text{w}}^{\left(2\right)}=0.125$ and *C*_b_ = 1.045 ⋅ 10^−5^; for state S4: $C_{\text{w}}^{\left(2\right)}=0.125$ and *C*_b_ = 1.5 ⋅ 10^−5^. The initial transient (10^5^ data points) was dismissed.

The next building block of our testbed is the modeling of a multistable system, in which transitions between more than two states (dynamical regimes) are not induced by a change of control parameters but occur in a self-induced manner. To this end, we consider a potential landscape with transitions between states that are mediated by the rare recurring, high-amplitude phenomena generated by the network of networks of FHN oscillators. We approximate the potential landscape *L*(*z*) by a succession of *N*_s_ potential wells (modeled as inverted Gaussian functions; *z* is the dynamical variable that describes the motion within the potential landscape), that mimic the basins of attraction of the *N*_s_ states of our multistable system (see upper part of Figure 2):

$$L\left(z\right)=\overset{N_{\text{s}}\;}{\underset{n=1}{\sum }}\frac{a_{n}^{\text{G}}}{\sqrt{2\pi {\left(\sigma_{n}^{\text{G}}\right)}^{2}}}exp\left(-\frac{{\left(z-\mu_{n}^{\text{G}}\right)}^{2}}{2{\left(\sigma_{n}^{\text{G}}\right)}^{2}}\right).$$ (4)

Here $a_{n}^{\text{G}}$ , $\mu_{n}^{\text{G}}$ , and $\sigma_{n}^{\text{G}}$ denote amplitude, mean and standard deviation of the *n*-th Gaussian function. Together with the distance Δ*z* between potential wells, these control parameters allow one to modify resilience of a system (see Mitra et al. (2015) and references therein). We couple the FHN-NoN’s mean field *M*(*t*) to the potential landscape and derive the equation which governs the motion of *z*:

$$\dot{z}\left(t\right)={\left.\dot{L}\left(z\right)\right|}_{z\left(t\right)}+\zeta \left(t\right)M\left(t\right),$$ (5)

where

$$M\left(t\right)=\frac{\Gamma \left(t\right)}{N_{\text{o}}\;}\overset{N_{\text{o}}\;}{\underset{i=1}{\sum }}x_{i}^{\left(1\right)}\left(C_{\text{w}}^{\left(1\right)},C_{\text{b}},t\right)+x_{i}^{\left(2\right)}\left(C_{\text{w}}^{\left(2\right)},C_{\text{b}},t\right)$$ (6)

and *ζ*(*t*) = (0.6 − 0.01*z*(*t*)) is a scaling factor. The second term on the r.h.s. of Eq. 5 enables the switching of the dynamics into different potential wells mediated by the high-amplitude phenomena generated by the FHN-NoN. This self-induced switching thus solely depends on the amplitude of the FHN-NoN’s mean field *M*(*t*) and is not mediated by a change of control parameters of the potential landscape. In order to guarantee distinguishable dynamics for the different potential wells, we change the coupling strengths $C_{\text{w}}^{\left(k\right)}$ and *C*_b_ once the mean field *M*(*t*) drives *z*(*t*) to overcome the local barrier between the respective wells. The asymmetric amplitude distribution (cf. Figure 1) of the mean field dominates the sequence in which states are switched. As an example, setting Γ(*t*) = + 1 *∀t* results in a sequence of state switches from the first to the last potential well. More complicated sequences of state switches can be achieved by setting Γ(*t*) (with $\left|\Gamma \left(t\right)\right|=1\;\forall t$ ) appropriately. For example, choosing the sign of a set of random numbers drawn from some distribution centered around zero results in a random sequence of state switches. The sign of Γ(*t*) may also be chosen depending on the current state of the system. In the upper part of Figure 2, we show for a 4-state system exemplary phase space representations of the averaged dynamical variables for each state. In the lower part, we show for two choices of Γ(*t*) exemplary time series of the dynamical variable *z*(*t*) of the potential landscape and of the averaged dynamical variable ${\bar{x}}^{\left(1\right)}\left(t\right)$ of sub-network 1.

There are alternative ways to model a potential landscape (Hänggi et al., 1990), for example using an *n*-th order polynomial. Advantages of using a succession of inverted Gaussian functions include the simple and intuitive way of adding further potential wells, thereby retaining the order of the wells. A disadvantage is the smooth barrier between potential wells, which may result in a rapid escape from a well once the above mentioned escape condition is fulfilled, and thus to short transition times (cf. Figure 2). This can be avoided by using, for example, fragmented barriers that can be constructed using the classic Cantor fractal construction process (see, e.g., Mandelbrot (1982); Omelchenko et al. (2015)). Such fragmented barriers may also mimic riddled basin of attractions (Alexander et al., 1992). Another way to achieve a non-smooth barrier would be adding e.g., colored noise to the potential landscape.

**FIGURE 3:**
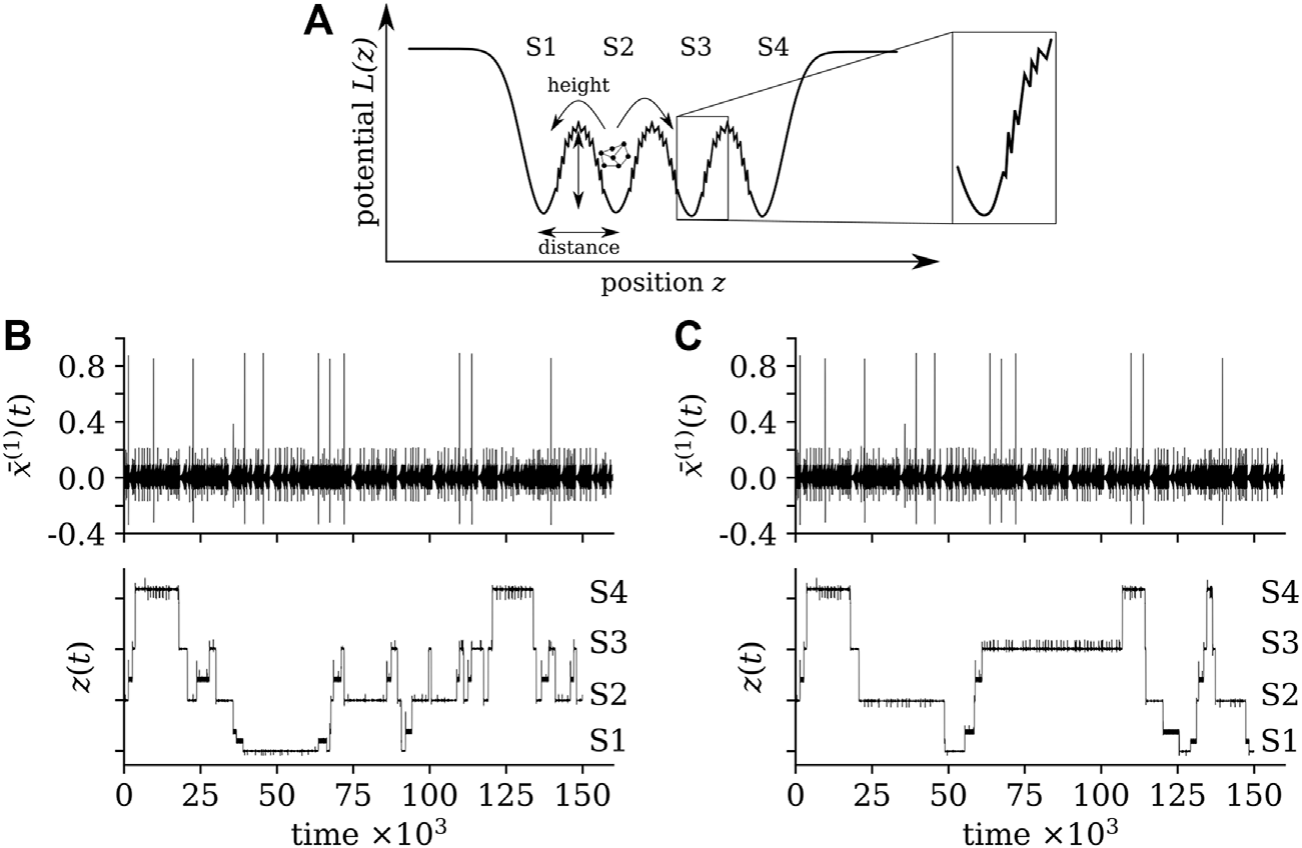
**(A)**: same potential landscape as in Figure 2 but with fragmented barriers, generated by using the classic Cantor fractal construction process. We bridge “gaps” in the barriers that result from the fractal construction process by inserting segments with adjustable steepness *α*. The fragmented barrier begins and ends outside of the local minimum of a potential well such that an amplitude-based transition can be induced from the potential well into the barrier. **(B,C)**: Exemplary time series of the averaged dynamical variable ${\bar{x}}^{\left(1\right)}\left(t\right)$ of the FHN-NoN and of the dynamical variable *z*(*t*) of the potential landscape.

**FIGURE 4:**
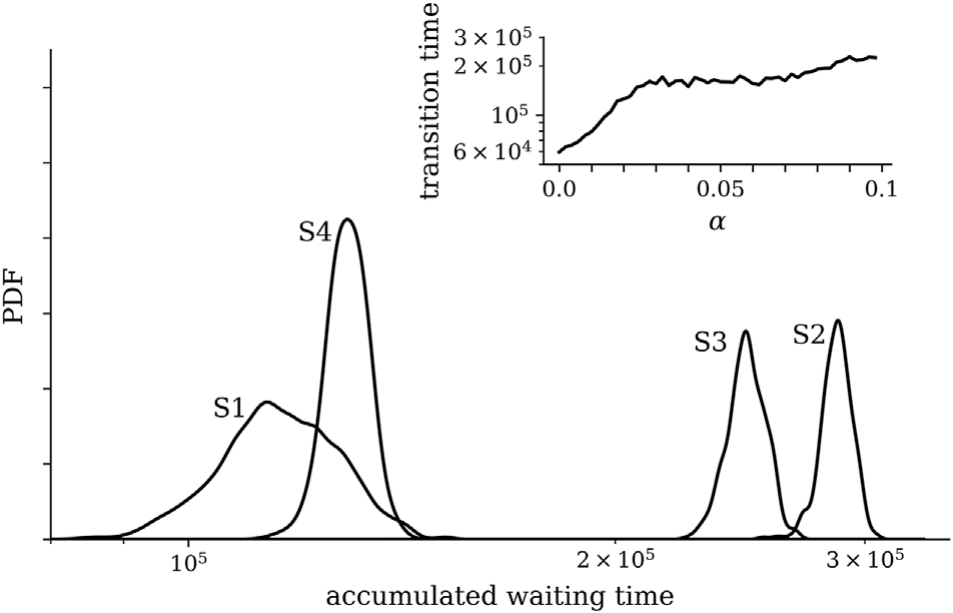
Distributions of accumulated waiting times of the FHN-NoN dynamics within the potential wells (states S1–S4) of the landscape with fragmented barriers shown in Figure 3 and for Γ(*t*) chosen state-dependent such that the system evolves alternating from the first to the last state and back. Distributions (kernel density estimates) were derived from 10 realizations with random initial conditions of oscillators and of the dynamical variable *z*(*t*) of the landscape. Other control parameters as in Figure 3. The larger waiting times within states S2 and S3 result from these states being visited, on average, more often than the other states as they each can be reached *via* two transitions. The inset shows the dependence of the average transition time between potential wells on the steepness *α* of the fragmented barrier. The average transition time was estimated over 20 realizations with different initial conditions. Lines are for eye-guidance only.

An example of a potential landscape with fragmented barriers is shown in Figure 3 along with time series of the averaged dynamical variable ${\bar{x}}^{\left(1\right)}\left(t\right)$ of sub-network 1 of the FHN-NoN and of the dynamical variable *z*(*t*) of the potential landscape. The inclusion of a fragmented barrier can be regarded as adding “intermediate states” that temporarily trap the system. Note that the dynamics within these intermediate states differs from the ones observed in the potential wells. Figure 4 provides a synopsis of the accumulated waiting times of the FHN-NoN dynamics within each state and demonstrates how the steepness of fragmented barriers impacts on the transition time between states.

### 2.3 Modifying the System’s Resilience: An Example

**FIGURE 5:**
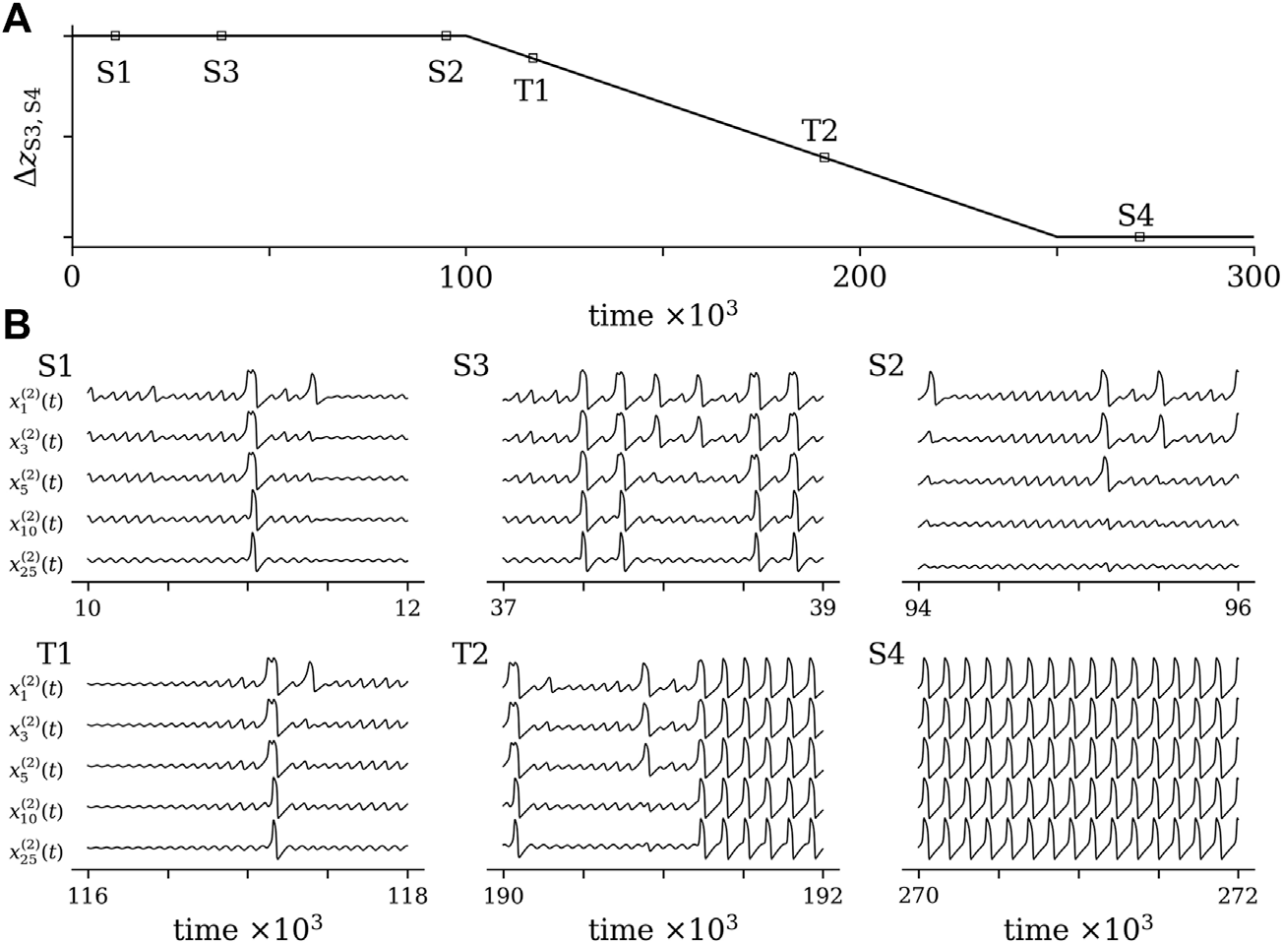
Schematic of a modification of resilience of a multistable system with three desired states (S1, S2, S3) and one undesired state (S4) **(A)** and excerpts of exemplary time series of system observables from various states **(B)**. Setting of control parameters of the potential landscape: *a* ^G^ = [36.5, 36.5, 36.5, 36.5]; *μ* ^G^ = [ − 18.3, − 6.2, 6.2, 22.3]; ${\left(\sigma^{\text{G}}\right)}^{2}=\left[12,12,12,5\right]$ . Coupling constant for all states: $C_{\text{w}}^{\left(1\right)}=0.115$ , for state S1: $C_{\text{w}}^{\left(2\right)}=0.116$ and *C*_b_ = 1.045 ⋅ 10^−5^; for state S2: $C_{\text{w}}^{\left(2\right)}=0.116$ and *C*_b_ = 1.425 ⋅ 10^−5^; for state S3: $C_{\text{w}}^{\left(2\right)}=0.125$ and *C*_b_ = 1.045 ⋅ 10^−5^; for state S4: $C_{\text{w}}^{\left(2\right)}=1.25$ and *C*_b_ = 1.5 ⋅ 10^−5^. Initial configuration at time interval $t\in \left[0,10^{5}\right)$ : Self-induced transitions are possible between states S1, S2, and S3 but a transition into the undesired state S4 is not possible. Starting at time *t* = 10^5^, the position $\mu_{4}^{\text{G}}$ is linearly decreased up to $\mu_{4}^{\text{G}}=19.3$ at time *t* = 2.5 ⋅ 10^-5^, which decreases the distance Δ*z*_S3,S4_ between S3 and S4. The lower part of the figure exemplifies excerpts of time series of the *x*-components of oscillators 1, 3, 5, 10. and 25 (increasing value of control parameter *b*_*i*_) of sub-network 2 at time intervals indicated in the upper part. The excerpts are labeled according to the system’s state, and T1 and T2 indicate excerpts from transitory phases.

As already mentioned above, several control parameters allow one to modify the resilience of our multistable system which is briefly illustrated in the following. We consider a system as in Figure 3 with three *desired* states (S1, S2, S3) representing its normal functioning and one *undesired* state (S4) representing an aberrant functioning. The parameters controlling the distance between wells S1, S2 and S3 as well as the height of the barriers between these wells are identical but differ from those of well S4. We allow for a state-dependent switching between states, and by gradually moving S4 closer to S3 (i.e., decreasing the distance Δ*z* between these states), we mimic a progressive loss of the system’s resilience. Since the height of the barrier between S3 and S4 is enlarged, the system is trapped in S4 once it enters this state. A schematic of this modification along with exemplary excerpts of time series of observables from some oscillators are presented in Figure 5.

Before closing this section, we briefly summarize the main aspects of our testbed that allow us to modify resilience of a multistable networked dynamical system in a controlled manner. Our testbed provides the means to simulate the dynamics of a multistable system with the help of a network of networks of FitzHugh-Nagumo oscillators coupled to a potential landscape that consists of a succession of a number of potential wells with smooth or fragmented barriers. Various control parameters allow one to generate distinguishable dynamics for each (desired or undesired) state, to adjust the waiting time of the system within each state, as well as the transition time between states. Our testbed also allows for a generation of time series of system observables, and these time series may serve as input to data-driven indicators of resilience.

## 3 An Exemplary Evaluation of a Data-Driven Indicator of Resilience

In the following, we utilize time series generated by our testbed for an exemplification of a data-driven indicator of resilience. Rings et al. (2019) proposed a time-series-based and non-perturbative approach to efficiently monitor *dynamical resistance*, an indicator of resilience of a networked dynamical system. The approach is fully data-driven since it takes into account the units’ individual signals only and consists of the following three central steps of analysis:

1 Probe with high temporal resolution the dynamical coupling structure between interacting system units;
2 identify dynamical regimes (here: states) from similar time-dependent coupling structures;
3 define dynamical resistance *R* as the minimum *distance* between all accessible dynamical regimes.

Step of analysis # 1: The dynamical coupling structure (the second term on the r.h.s. of Eq. 1; coupling strength, coupling structure, and coupling function) can be probed with bivariate time-series-analysis techniques developed in statistics, nonlinear dynamics, information and synchronization theory as well as in statistical physics (Pikovsky et al., 2001; Kantz and Schreiber, 2003; Pereda et al., 2005; Hlaváčková-Schindler et al., 2007; Marwan et al., 2007; Stankovski et al., 2017; Tabar, 2019). Here we use three widely-used techniques, namely the zero- and maximum-lag cross-correlation (Brillinger, 1981; Rosenblum et al., 1997) as well as the (normalized) mutual information (Kraskov et al., 2004). These techniques allow one to estimate the similarity/interdependence *ρ*_*uv*_ between pairs of time series $\left\{u\right\}$ and $\left\{v\right\}$ each of length *T* (with *T* much smaller than the total observation time). If appropriately normalized, *ρ*_*uv*_ assumes values between 0 and 1, indicating either complete independence or complete dependence. We use a sliding window approach to calculate *ρ*_*uv*_ between all pairs of units in a time-resolved manner which results in a temporal sequence of interaction matrices *ρ*.

Step of analysis # 2: In order to identify dynamical regimes, one can define similarity between two interaction matrices *ρ* (*t*_*l*_) and *ρ* (*t*_*m*_) at times *t*_*l*_ and *t*_*m*_ as *ξ* (*t*_*l*_, *t*_*m*_) ≡‖*ρ* (*t*_*l*_) − *ρ* (*t*_*m*_)‖, where ‖… ‖ denotes the Euclidean norm (Münnix et al., 2012). The similarity matrix *ξ*—estimated for all times *t*_*m*_ and *t*_*l*_—then contains pertinent information about the system’s dynamics, and recurrent patterns in the similarity matrix indicate dynamical regimes (Marwan et al., 2007). In order to identify these regimes and their number, Rings et al. (2019) proposed to use a time-resolved hierarchical clustering analysis of coupling structures in an abstract space spanned by all pairwise interactions. For our investigations, we use a k-means algorithm (MacQueen, 1967) given that the number of different dynamical regimes (clusters k = *N*_s_) is known a priori.

Step of analysis # 3: The minimum Euclidean separation between cluster centroids is taken as the minimum distance between dynamical regimes and is an estimate for dynamical resistance *R*: the larger this distance between regimes the higher is the capacity of a system to absorb disturbances and to reorganize while undergoing dynamical changes so as to still retain essentially the same functionality.

For our investigations, we consider the example from Section 2.3 and simulate a gradual loss of resilience of the system, which we assume to result from a “perturbation” mediated by the undesired state. We again incrementally decrease the distance Δ*z*_S3,S4_ between states S3 and S4, and for each increment we record time series of observables of each FHN oscillator for 10^6^ time steps thereby starting from identical initial conditions of each oscillator. For our analyses, we use the oscillators’ *x*-components from sub-network 2 that we observe using the identity as measurement function.

**FIGURE 6:**
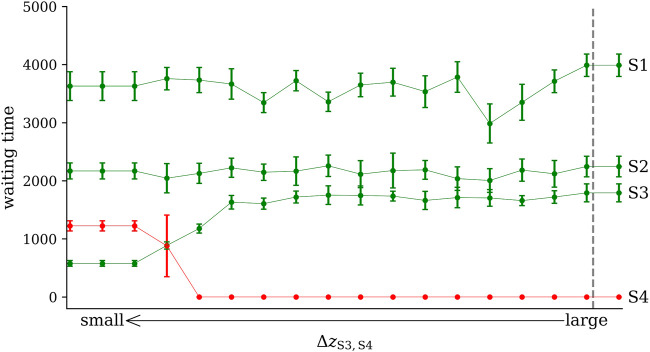
Impact of decreasing the distance Δ*z*_S3,S4_ between states S3 and S4 on the waiting times in each of the four states (S1–S4). Medians and variances (lengths of error bars) obtained from 10 realizations of the simulation setup. A zero value of the waiting time of S4 indicates that this state is never reached because it is too far away from S3. The vertical dashed line indicates onset of perturbation. Other lines are for eye-guidance only.

**FIGURE 7:**
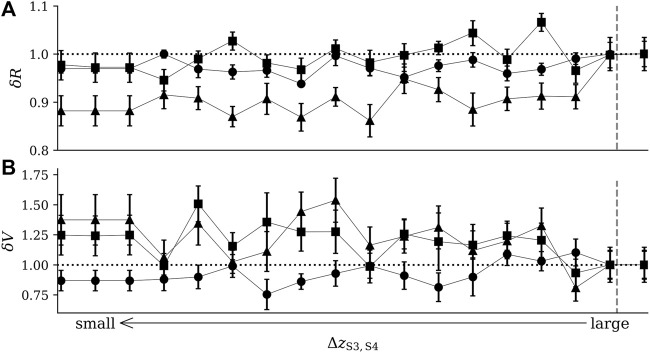
Relative change of dynamical resistance [*δR*; **(A)**] and of area between cluster centroids [*δV*; **(B)**] upon decreasing the distance Δ*z*_S3,S4_ between states S3 and S4. Dynamical resistance *R* estimated with mutual information (filled circles), zero-lag cross-correlation (filled triangles), and maximum-lag cross-correlation (filled squares). Area *V* between cluster centroids calculated from the distances between the three cluster centroids representing states S1–S3 (symbols as above). Data normalized to the respective values for large Δ*z*_S3,S4_. Medians and variances (lengths of error bars) obtained from 10 realizations of the simulation setup. The vertical dashed line indicates onset of perturbation. Other lines are for eye-guidance only.

In Figure 6, we show how the shortening of the distance Δ*z*_S3,S4_ impacts on the waiting times within each state. As expected, the median waiting time within state S4 increased upon decreasing Δ*z*_S3,S4_, while within state S3 the median waiting time gradually decreased. Waiting times within states S1 and S2 remained largely unaffected by the perturbation. In the upper part of Figure 7, we summarize our findings for dynamical resistance *R*. Depending on the bivariate time-series-analysis technique employed to estimate *R*, we observe the initial resilience of the system to be diminished by about 10% as S4 gets closer to S3. Our interpretation of this loss of resilience due to a “perturbation” mediated by an undesired state is further corroborated by the distinct increased area between cluster centroids reflecting a deformation of the initial configuration of the system’s dynamical regimes (lower part of Figure 7).

## 4 Conclusion

We develop a testbed that allows one to modify resilience of a multistable networked dynamical system in a controlled manner and to generate time series of observables that may be used to evaluate the suitability of data-driven indicators of resilience. The testbed presented here was designed in such a way that it provides a means—with the help of an adjustable potential landscape, sufficiently many and, more importantly, contextual control parameters—to simulate a multistable system with a number of system states and with self-induced switching between them as well as to simulate distinguishable dynamics for each state. Waiting times within states are sufficiently long to allow data-driven indicators of resilience to reliably characterize the different states. With the inclusion of fragmented barriers between potential wells, transition times between states can be preset. We here considered a potential landscape—consisting of a succession of adjustable wells—driven by the rich dynamics of a network of networks of diffusively coupled FitzHugh-Nagumo (FHN) oscillators (Rydin Gorjão et al., 2018). The network’s dynamics is chaotic and can exhibit different dynamical patterns such as low-amplitude oscillations, nonlinear waves, and rare recurring high-amplitude phenomena (Ansmann et al., 2013). The network can also exhibit self-induced switchings between these patterns (states) without a change of control parameters (Ansmann et al., 2016). Our testbed allows for short computation times and can modify resilience during run time. As an example, the generation of time series of observables of a fully connected network of two networks of 50 FHN oscillators with 10^6^ data points each requires about 3 min on a 64-bit architecture with a single CPU at 2.2 GHz.

Using time series data generated by our testbed for a multistable system gradually perturbed by an undesired state, we performed an exemplary evaluation of a data-driven indicator of resilience of a networked dynamical system (Rings et al., 2019). Our findings indicate that this indicator—dynamical resistance *R*—appears to be capable of tracking changes in resilience, at least to some extent and for the scenario considered here. Nevertheless, findings also indicate that its performance appears to depend on the bivariate time-series-analysis technique employed to characterize couplings between system units. Future studies would need to address the question as to which extent the influence of a unit’s self-dynamics can be neglected when estimating resilience of a networked dynamical system. In addition, future studies would need to tackle the largely unsolved issue of how to reliably interpret findings obtained with data-driven indicators particularly with respect to Holling’s definition of resilience.

We foresee various extensions to our testbed, also in view of evaluating other data-driven indicators. For example, one may consider other configurations of the potential landscape, other models of networked dynamics, coupling and measurement functions that are of relevance for a given research field. Time-dependent control parameters [see, e.g., Nicolis and Nicolis (2014); O’Regan and Burton (2018)] for both the network dynamics and for the potential landscape will introduce various non-stationarities, thus bringing our testbed closer to natural systems. At a similar token, the introduction of stochasticity (Freidlin and Wentzell, 1984; Arnold, 1998) into our testbed may allow for various noise-related phenomena such as noise-induced transitions (Horsthemke and Lefever, 1984), stochastic resonance (Gammaitoni et al., 1998), or noise-induced tipping (Ritchie and Sieber, 2017; Wunderling et al., 2021). Their time-series-analysis-based investigation in networked dynamical system may, however, require more refined and better adapted analysis techniques (Rydin Gorjão et al., 2019, 2021; Tabar, 2019; Aslim et al., 2021).

## Data Availability

The raw data supporting the conclusion of this article will be made available by the authors, without undue reservation. The code of this work has been provided as the open source software package resiland written in the programming language Python. It is freely available on github under the: https://github.com/tobfischer/resiland.

## References

[B1] Alexander J. C. Yorke J. A. You Z. Kan I. (1992). Riddled Basins. Int. J. Bifurcation Chaos 02, 795–813. 10.1142/s0218127492000446

[B2] Ansmann G. Karnatak R. Lehnertz K. Feudel U. (2013). Extreme Events in Excitable Systems and Mechanisms of Their Generation. Phys. Rev. E 88, 052911. 10.1103/PhysRevE.88.052911 24329335

[B3] Ansmann G. Lehnertz K. Feudel U. (2016). Self-induced Switchings between Multiple Space-Time Patterns on Complex Networks of Excitable Units. Phys. Rev. X 6, 011030. 10.1103/PhysRevX.6.011030

[B4] Ansmann G. (2018). Efficiently and Easily Integrating Differential Equations with JiTCODE, JiTCDDE, and JiTCSDE. Chaos 28, 043116. 10.1063/1.5019320 31906633

[B5] Arnold L. (1998). Random Dynamical Systems. Berlin: Springer.

[B6] Ashwin P. Wieczorek S. Vitolo R. Cox P. (2012). Tipping Points in Open Systems: Bifurcation, Noise-Induced and Rate-dependent Examples in the Climate System. Phil. Trans. R. Soc. A. 370, 1166–1184. 10.1098/rsta.2011.0306 22291228

[B7] Aslim E. Rings T. Zabawa L. Lehnertz K. (2021). Enhancing the Accuracy of a Data-Driven Reconstruction of Bivariate Jump-Diffusion Models with Corrections for Higher Orders of the Sampling Interval. J. Stat. Mech. 2021, 033406. 10.1088/1742-5468/abe59a

[B8] Barabási A. Posfai M. (2016). Network Science. 1st edn. Cambridge, UK: Cambridge University Press.

[B9] Benettin G. Galgani L. Giorgilli A. Strelcyn J.-M. (1980a). Lyapunov Characteristic Exponents for Smooth Dynamical Systems and for Hamiltonian Systems; a Method for Computing All of Them. Part 1: Theory. Meccanica 15, 9–20. 10.1007/BF02128236

[B10] Benettin G. Galgani L. Giorgilli A. Strelcyn J.-M. (1980b). Lyapunov Characteristic Exponents for Smooth Dynamical Systems and for Hamiltonian Systems; a Method for Computing All of Them. Part 2: Numerical Application. Meccanica 15, 21–30. 10.1007/BF02128237

[B11] Boettiger C. Hastings A. (2013). No Early Warning Signals for Stochastic Transitions: Insights from Large Deviation Theory. Proc. R. Soc. B. 280, 20131372. 10.1098/rspb.2013.1372 PMC3730602 23843397

[B12] Bonhoeffer K. F. (1948). Activation of Passive Iron as a Model for the Excitation of Nerve. J. Gen. Physiol. 32, 69–91. 10.1085/jgp.32.1.69 18885679 PMC2213747

[B13] Brillinger D. (1981). Time Series: Data Analysis and Theory. San Francisco, USA: Holden Day.

[B14] Chernihovskyi A. Lehnertz K. (2007). Measuring Synchronization with Nonlinear Excitable Media. Int. J. Bifurcation Chaos 17, 3425–3429. 10.1142/s0218127407019159

[B15] Clements C. F. McCarthy M. A. Blanchard J. L. (2019). Early Warning Signals of Recovery in Complex Systems. Nat. Commun. 10, 1681. 10.1038/s41467-019-09684-y 30975997 PMC6459826

[B16] Dai L. Vorselen D. Korolev K. S. Gore J. (2012). Generic Indicators for Loss of Resilience before a Tipping point Leading to Population Collapse. Science 336, 1175–1177. 10.1126/science.1219805 22654061

[B17] Dakos V. Carpenter S. R. van Nes E. H. Scheffer M. (2015). Resilience Indicators: Prospects and Limitations for Early Warnings of Regime Shifts. Phil. Trans. R. Soc. B 370, 20130263. 10.1098/rstb.2013.0263

[B18] Diks C. Hommes C. Wang J. (2019). Critical Slowing Down as an Early Warning Signal for Financial Crises? Empir. Econ. 57, 1201–1228. 10.1007/s00181-018-1527-3

[B19] Ditlevsen P. D. Johnsen S. J. (2010). Tipping Points: Early Warning and Wishful Thinking. Geophys. Res. Lett. 37, L19703. 10.1029/2010gl044486

[B20] FitzHugh R. (1961). Impulses and Physiological States in Theoretical Models of Nerve Membrane. Biophysical J. 1, 445–466. 10.1016/S0006-3495(61)86902-6 PMC1366333 19431309

[B21] Freidlin M. I. Wentzell A. D. (1984). Random Perturbations of Dynamical Systems. New York: Springer

[B22] Gammaitoni L. Hänggi P. Jung P. Marchesoni F. (1998). Stochastic Resonance. Rev. Mod. Phys. 70, 223–287. 10.1103/RevModPhys.70.223

[B23] Gerster M. Berner R. Sawicki J. Zakharova A. Škoch A. Hlinka J. (2020). FitzHugh–Nagumo Oscillators on Complex Networks Mimic Epileptic-Seizure-Related Synchronization Phenomena. Chaos: Interdiscip. J. Nonlinear Sci. 30, 123130. 10.1063/5.0021420 33380049

[B24] Glass L. Hunter P. McCulloch A. (1991). Theory of Heart. New York: Springer. 10.1007/978-1-4612-3118-9

[B25] Hänggi P. Talkner P. Borkovec M. (1990). Reaction-rate Theory: Fifty Years after Kramers. Rev. Mod. Phys. 62, 251. 10.1103/revmodphys.62.251

[B26] Hagemann A. Wilting J. Samimizad B. Mormann F. Priesemann V. (2021). Assessing Criticality in Pre-seizure Single-Neuron Activity of Human Epileptic Cortex. Plos Computat. Biol. 17, e1008773. 10.1371/journal.pcbi.1008773 PMC7971851 33684101

[B27] Hernández-Navarro L. Faci-Lázaro S. Orlandi J. G. Feudel U. Gómez-Gardeñes J. Soriano J. (2021). Noise-driven Amplification Mechanisms Governing the Emergence of Coherent Extreme Events in Excitable Systems. Phys. Rev. Res. 3, 023133. 10.1103/physrevresearch.3.023133

[B28] Hlaváčková-Schindler K. Paluš M. Vejmelka M. Bhattacharya J. (2007). Causality Detection Based on Information-Theoretic Approaches in Time Series Analysis. Phys. Rep. 441, 1–46. 10.1016/j.physrep.2006.12.004

[B29] Holling C. S. Goldberg M. A. (1971). Ecology and Planning. J. Am. Planners 37, 221–230. 10.1080/01944367108977962

[B30] Horsthemke W. Lefever R. (1984). Noise-Induced Transitions. Theory and Applications in Physics, Chemistry and Biology. Berlin: Springer.

[B31] Kantz H. Schreiber T. (2003). Nonlinear Time Series Analysis. 2nd edn. Cambridge, UK: Cambridge University Press. 10.1017/CBO9780511755798

[B32] Karnatak R. Ansmann G. Feudel U. Lehnertz K. (2014). Route to Extreme Events in Excitable Systems. Phys. Rev. E 90, 022917. 10.1103/PhysRevE.90.022917 25215809

[B33] Koch C. (1999). Biophysics of Computation: Information Processing in Single Neurons. New York: Oxford University Press Computational Neuroscience.

[B34] Kraskov A. Stögbauer H. Grassberger P. (2004). Estimating Mutual Information. Phys. Rev. E 69, 066138. 10.1103/PhysRevE.69.066138 15244698

[B35] Kuehn C. Zschaler G. Gross T. (2015). Early Warning Signs for Saddle-Escape Transitions in Complex Networks. Sci. Rep. 5, 13190. 10.1038/srep13190 26294271 PMC4544003

[B36] Kuehn C. (2011). A Mathematical Framework for Critical Transitions: Bifurcations, Fast-Slow Systems and Stochastic Dynamics. Physica D 240, 1020–1035. 10.1016/j.physd.2011.02.012

[B37] Lehnertz K. Bröhl T. Rings T. (2020). The Human Organism as an Integrated Interaction Network: Recent Conceptual and Methodological Challenges. Front. Physiol. 11, 1694. 10.3389/fphys.2020.598694 PMC7779628 33408639

[B38] Lenton T. Livina V. Dakos V. Van Nes E. Scheffer M. (2012). Early Warning of Climate Tipping Points from Critical Slowing Down: Comparing Methods to Improve Robustness. Phil. Trans. Roy. Soc. A: Math. Phys. Eng. Sci. 370, 1185–1204. 10.1098/rsta.2011.0304 PMC3261433 22291229

[B39] Lyapunov A. M. (1892). The General Problem Of Stability Of Motion (In Russian) Doctoral dissertation Kharkov: University of Kharkov, Kharkov Mathematical Society.

[B40] MacQueen J. B. (1967). “Some Methods for Classification and Analysis of Multivariate Observations,” in Fifth Berkeley Symposium on Mathematical Statistics and Probability, June 21–July 18, 1965 and December 27, 1965–January 7, 1966. Editors Cam M. L. Neyman J. (Berkeley, USA: Statistical Laboratory of the University of California), 281–297.

[B41] Mandelbrot B. B. (1982). The Fractal Geometry of Nature. San Francisco: Freeman.:

[B42] Marconi M. Métayer C. Acquaviva A. Boyer J. Gomel A. Quiniou T. (2020). Testing Critical Slowing Down as a Bifurcation Indicator in a Low-Dissipation Dynamical System. Phys. Rev. Lett. 125, 134102. 10.1103/physrevlett.125.134102 33034502

[B43] Marwan N. Romano M. C. Thiel M. Kurths J. (2007). Recurrence Plots for the Analysis of Complex Systems. Phys. Rep. 438, 237–329. 10.1016/j.physrep.2006.11.001

[B44] Masoliver M. Malik N. Schöll E. Zakharova A. (2017). Coherence Resonance in a Network of FitzHugh-Nagumo Systems: Interplay of Noise, Time-Delay, and Topology. Chaos: Interdiscip. J. Nonlinear Sci. 27, 101102. 10.1063/1.5003237 29092412

[B45] Meyer K. (2016). A Mathematical Review of Resilience in Ecology. Nat. Resour. Model. 29, 339–352. 10.1111/nrm.12097

[B46] Mitra C. Kurths J. Donner R. V. (2015). An Integrative Quantifier of Multistability in Complex Systems Based on Ecological Resilience. Sci. Rep. 5, 16196. 10.1038/srep16196 26537459 PMC4633666

[B47] Münnix M. C. Shimada T. Schäfer R. Leyvraz F. Seligman T. H. Guhr T. (2012). Identifying States of a Financial Market. Sci. Rep. 2, 644. 10.1038/srep00644 22966419 PMC3437514

[B48] Nagumo J.-I. S. Arimoto S. Yoshizawa S. (1962). An Active Pulse Transmission Line Simulating Nerve Axon. Proc. IRE 50, 2061–2070. 10.1109/jrproc.1962.288235

[B49] Nicolis C. Nicolis G. (2014). Dynamical Responses to Time-dependent Control Parameters in the Presence of Noise: a normal Form Approach. Phys. Rev. E 89, 022903. 10.1103/PhysRevE.89.022903 25353541

[B50] O’Keeffe P. E. Wieczorek S. (2020). Tipping Phenomena and Points of No Return in Ecosystems: beyond Classical Bifurcations. SIAM J. Appl. Dyn. Syst. 19, 2371–2402. 10.1137/19m1242884

[B51] O’Regan S. M. Burton D. L. (2018). How Stochasticity Influences Leading Indicators of Critical Transitions. Bull. Math. Biol. 80, 1630–1654. 10.1007/s11538-018-0429-z 29713924

[B52] Omelchenko I. Provata A. Hizanidis J. Schöll E. Hövel P. (2015). Robustness of Chimera States for Coupled FitzHugh-Nagumo Oscillators. Phys. Rev. E 91, 022917. 10.1103/PhysRevE.91.022917 25768579

[B53] Pereda E. Quian Quiroga R. Bhattacharya J. (2005). Nonlinear Multivariate Analysis of Neurophysiological Signals. Prog. Neurobiol. 77, 1–37. 10.1016/j.pneurobio.2005.10.003 16289760

[B54] Pikovsky A. S. Rosenblum M. G. Kurths J. (2001). Synchronization: A Universal Concept in Nonlinear Sciences. Cambridge, UK: Cambridge University Press.

[B55] Plotnikov S. Lehnert J. Fradkov A. Schöll E. (2016). Synchronization in Heterogeneous FitzHugh-Nagumo Networks with Hierarchical Architecture. Phys. Rev. E 94, 012203. 10.1103/PhysRevE.94.012203 27575119

[B56] Quinlan A. E. Berbés-Blázquez M. Haider L. J. Peterson G. D. (2016). Measuring and Assessing Resilience: Broadening Understanding through Multiple Disciplinary Perspectives. J. Appl. Ecol. 53, 677–687. 10.1111/1365-2664.12550

[B57] Ramlow L. Sawicki J. Zakharova A. Hlinka J. Claussen J. C. Schöll E. (2019). Partial Synchronization in Empirical Brain Networks as a Model for Unihemispheric Sleep. EPL (Europhys. Lett.) 126, 50007. 10.1209/0295-5075/126/50007

[B58] Rings T. Ansmann G. Lehnertz K. (2017). How Important Are Hubs for the Generation of Extreme Events in Networks of Excitable Units? Eur. Phys. J.-Spec. Top. 226, 1963–1970. 10.1140/epjst/e2017-70021-3

[B59] Rings T. Mazarei M. Akhshi A. Geier C. Tabar M. R. R. Lehnertz K. (2019). Traceability and Dynamical Resistance of Precursor of Extreme Events. Sci. Rep. 9, 1744. 10.1038/s41598-018-38372-y 30741977 PMC6370838

[B60] Ritchie P. Sieber J. (2017). Probability of Noise-And Rate-Induced Tipping. Phys. Rev. E 95, 052209. 10.1103/PhysRevE.95.052209 28618548

[B61] Rocşoreanu C. Georgescu A. Giurgiţeanu N. (2000). The FitzHugh–Nagumo Model: Bifurcation and Dynamics. Dordrecht: Kluwer Academic Publishers.

[B62] Romero-Ortuño R. Martínez-Velilla N. Sutton R. Ungar A. Fedorowski A. Galvin R. (2021). Network Physiology in Aging and Frailty: The Grand Challenge of Physiological Reserve in Older Adults. Front. Netw. Physiol. 1, 712430. 10.3389/fnetp.2021.712430 PMC10012993 36925570

[B63] Rosenblum M. G. Pikovsky A. S. Kurths J. (1997). From Phase to Lag Synchronization in Coupled Chaotic Oscillators. Phys. Rev. Lett. 78, 4193–4196. 10.1103/physrevlett.78.4193

[B64] Rydin Gorjão L. Saha A. Ansmann G. Feudal U. Lehnertz K. (2018). Complexity and Irreducibility of Dynamics on Networks of Networks. Chaos: Interdiscipilinary J. Nonlinear Sci. 28, 106306. 10.1063/1.5039483 30384647

[B65] Rydin Gorjão L. Heysel J. Lehnertz K. Tabar M. R. R. (2019). Analysis and Data-Driven Reconstruction of Bivariate Jump-Diffusion Processes. Phys. Rev. E 100, 062127. 10.1103/PhysRevE.100.062127 31962437

[B66] Rydin Gorjão L. Witthaut D. Lehnertz K. Lind P. G. (2021). Arbitrary-order Finite-Time Corrections for the Kramers–Moyal Operator. Entropy 23, 517. 10.3390/e23050517 33923154 PMC8146575

[B67] Saha A. Feudel U. (2017). Extreme Events in FitzHugh-Nagumo Oscillators Coupled with Two Time Delays. Phys. Rev. E 95, 062219. 10.1103/PhysRevE.95.062219 28709240

[B68] Scheffer M. Bascompte J. Brock W. A. Brovkin V. Carpenter S. R. Dakos V. (2009). Early-warning Signals for Critical Transitions. Nature 461, 53–59. 10.1038/nature08227 19727193

[B69] Scheffer M. Bolhuis J. E. Borsboom D. Buchman T. G. Gijzel S. M. Goulson D. (2018). Quantifying Resilience of Humans and Other Animals. Proc. Natl. Acad. Sci. (U.S.A.) 115, 11883–11890. 10.1073/pnas.1810630115 30373844 PMC6255191

[B70] Schoenmakers S. Feudel U. (2021). A Resilience Concept Based on System Functioning: A Dynamical Systems Perspective. Chaos: Interdiscip. J. Nonlinear Sci. 31, 053126. 10.1063/5.0042755 34240958

[B71] Stankovski T. Pereira T. McClintock P. V. E. Stefanovska A. (2017). Coupling Functions: Universal Insights into Dynamical Interaction Mechanisms. Rev. Mod. Phys. 89, 045001. 10.1103/RevModPhys.89.045001 PMC6834002 31656134

[B72] Tabar M. R. R. (2019). Analysis and Data-Based Reconstruction of Complex Nonlinear Dynamical Systems: Using the Methods of Stochastic Processes. Cham-Switzerland: Springer. 10.1007/978-3-030-18472-8:

[B73] van der Bolt B. van Nes E. H. Scheffer M. (2021). No Warning for Slow Transitions. J. Roy. Soc. Interf. 18, 20200935. 10.1098/rsif.2020.0935 PMC8086860 33784883

[B74] van der Pol B. van der Mark J. (1928). The Heartbeat Considered as a Relaxation Oscillation, and an Electrical Model of the Heart. Phil. Mag. 7 (6), 763. 10.1080/14786441108564652

[B75] Vanselow A. Wieczorek S. Feudel U. (2019). When Very Slow Is Too Fast-Collapse of a Predator-Prey System. J. Theor. Biol. 479, 64–72. 10.1016/j.jtbi.2019.07.008 31302207

[B76] Walker B. Holling C. S. Carpenter S. Kinzig A. (2004). Resilience, Adaptability and Transformability in Social–Ecological Systems. Ecol. Soc. 9, 5. 10.5751/es-00650-090205

[B77] Weinans E. Quax R. van Nes E. H. van de Leemput I. A. (2021). Evaluating the Performance of Multivariate Indicators of Resilience Loss. Sci. Rep. 11, 9148. 10.1038/s41598-021-87839-y 33911086 PMC8080839

[B78] Wilkat T. Rings T. Lehnertz K. (2019). No Evidence for Critical Slowing Down Prior to Human Epileptic Seizures. Chaos: Interdiscip. J. Nonlinear Sci. 29, 091104. 10.1063/1.5122759 31575122

[B79] Wunderling N. Krönke J. Wohlfarth V. Kohler J. Heitzig J. Staal A. (2021). Modelling Nonlinear Dynamics of Interacting Tipping Elements on Complex Networks: the PyCascades Package. Eur. Phys. J. Spec. Top. 230, 3163–3176. 10.1140/epjs/s11734-021-00155-4

